# Thermal Response of Laboratory Rats (*Rattus norvegicus*) during the Application of Six Methods of Euthanasia Assessed by Infrared Thermography

**DOI:** 10.3390/ani13182820

**Published:** 2023-09-05

**Authors:** Adriana Domínguez-Oliva, Ismael Hernández-Ávalos, Adriana Olmos-Hernández, Juan Villegas-Juache, Antonio Verduzco-Mendoza, Daniel Mota-Rojas

**Affiliations:** 1 Master in Science Program “Maestría en Ciencias Agropecuarias”, Xochimilco Campus, Universidad Autónoma Metropolitana, Mexico City 04960, Mexico; 2 Neurophysiology of Pain, Behavior and Assessment of Welfare in Domestic Animals, DPAA, Xochimilco Campus, Universidad Autónoma Metropolitana (UAM), Mexico City 04960, Mexico; 3 Clinical Pharmacology and Veterinary Anesthesia, Biological Sciences Department, Facultad de Estudios Superiores Cuautitlán, Universidad Nacional Autónoma de México, Cuautitlán Izcalli 54714, Mexico; 4 Division of Biotechnology—Bioterio and Experimental Surgery, Instituto Nacional de Rehabilitación-Luis Guillermo Ibarra Ibarra (INR-LGII), Mexico City 14389, Mexico; 5 Bioterio and Experimental Surgery, Instituto Nacional de Cardiología Ignacio Chávez, Mexico City 14080, Mexico

**Keywords:** rodents, infrared thermography, pentobarbital, decapitation, CO_2_, isoflurane, euthanasia, refinement, welfare

## Abstract

**Simple Summary:**

The present study aimed to assess the infrared thermal response of laboratory rats (*Rattus norvegicus*) during the application of six euthanasia methods (injectable, inhalational, and physical) to determine the method that prevents or diminishes the stress response. The surface temperature was assessed in four thermal windows: ocular (T°_ocu_), auricular (T°_ear_), interscapular (T°_dor_), and caudal (T°_tai_). The results showed that inhalant methods (CO_2_ and isoflurane) had temperature alterations that could be suggestive of a marked stress response, in contrast to other methods such as pentobarbital, decapitation, and xylazine + ketamine. In conclusion, according to the thermal response of the rats, it is suggested that CO_2_ and isoflurane might cause distress and this needs to be considered when selecting these techniques as the method of euthanasia for laboratory rats.

**Abstract:**

Refinement is one of the principles aiming to promote welfare in research animals. The techniques used during an experimental protocol, including euthanasia selection, must prevent and minimize suffering. Although the current euthanasia methods applied to laboratory rodents are accepted, the controversial findings regarding the potential stress/distress they can cause is a field of research. The objective was to assess the thermal response of Wistar rats during various euthanasia methods using infrared thermography (IRT) to determine the method that prevents or diminishes the stress response and prolonged suffering. Pentobarbital (G_1_), CO_2_ (G_2_), decapitation (G_3_), isoflurane (G_4_), ketamine + xylazine (G_5_), and ketamine + CO_2_ (G_6_) were evaluated at five evaluation times with IRT to identify changes in the surface temperature of four anatomical regions: ocular (T°_ocu_), auricular (T°_ear_), interscapular (T°_dor_), and caudal (T°_tai_). Significant differences (*p* < 0.05) were found in G_2_ and G_4_, registering temperature increases from the administration of the drug to the cessation of respiratory rate and heart rate. Particularly, isoflurane showed a marked thermal response in T°_ocu_, T°_ear_, T°_dor_, and T°_tai_, suggesting that, in general, inhalant euthanasia methods induce stress in rats and that isoflurane might potentially cause distress, an effect that must be considered when deciding humane euthanasia methods in laboratory rodents.

## 1. Introduction

The use of animals is a key element for improvements in biomedical science [1,2], where rats and mice represent 87–98% of the total of species used in the scientific community [3,4]. The potential pain and stress that laboratory animals might experience is highly controversial [5]. Ethical animal research necessitates the selection of suitable euthanasia methods to minimize pain and distress, as proposed by the National Centre for the Replacement, Refinement, and Reduction of Animals in Research [6], not only to provide welfare but also to ensure the quality of results. These initiatives need be applied not only during the life of research animals, but also during the application of euthanasia methods with the aim of providing humane endpoints [7].

Currently, there is a debate around the euthanasia methods that are approved by the American Veterinary Medicine Association (AVMA). Injectable drugs (e.g., barbiturates and general anesthetics), inhalant agents (e.g., CO_2_ and isoflurane), and physical methods (e.g., decapitation and cervical dislocation) are recognized as acceptable techniques to induce a humane death without suffering [8]. However, some methods are under discussion since studies have shown potential adverse effects during their application. For example, inhalation of CO_2_ is aversive for rats [9] and induces bradycardia and potential anxiety due to hypoxia before loss of consciousness [10,11]. Moreover, CO_2_ forms carbonic acid and induces the activation of pain receptors [12]. Nonetheless, systematic reviews have contrasting results regarding the suitability of CO_2_ and its potential distress [13]. Isoflurane is considered an alternative to CO_2_ euthanasia. However, it is known as an aversive agent to rats for its mild pungency [14,15].

On the other hand, the administration of injectable pentobarbital has been associated with pain-related behaviors (e.g., writhing and back arching) due to intraperitoneal (IP) irritation [16,17,18], while the combination of xylazine and ketamine, although a common anesthetic protocol, demonstrates limited action as a euthanasic agent. However, high Mouse Grimace Scale scores and anxiety-related behaviors were found after repeated doses of the combination [19]. Moreover, Wellington et al. [20] found that IP administration of ketamine + xylazine to rats caused acute muscle and tissue necrosis, poor tolerance, and pain/discomfort behavioral reactions. In the case of decapitation, this procedure leads to the question of whether brain activity is present immediately after the procedure or not, as well as whether changes in the electroencephalogram (EEG) are associated with nociception during the first 15 s (s) following decapitation [21], as determined in rats by Derr [22] who reported that EEGs during the first 2.7 s after decapitation might indicate conscious awareness of pain and distress.

The refinement of procedures performed in research animals includes the implementation of non-invasive tools to assess their welfare without causing additional stress. Infrared thermography (IRT) is a technique that detects surface temperature changes as a neuroendocrine response of the Sympathetic Nervous System (SNS) after stressful/distressful and painful events [23,24]. Stress—known as the reaction of the organism when its homeostasis or psychological well-being is perturbated—and distress—a negative and aversive state when the organism cannot adapt or return to homeostasis [25]—activates two main systems: the hypothalamic–pituitary–adrenal (HPA) and the *locus coeruleus* sympathetic adrenomedullary (SAM) axes [26]. Both axes lead to the release of glucocorticoids and catecholamines, as well as the physiological changes required to adjust homeostasis [27], including alterations in body temperature and microcirculation. Therefore, temperature variations have been used as a stress-related marker in animals, as stress may cause central hyperthermia and peripheral reduction of the temperature due to vasoconstriction [28]. 

IRT detects these vasomotor changes as a difference in the amount of dissipated heat in different anatomical regions, where heat exchange is facilitated through the arteriovenous anastomosis and peripheral blood vessels, also called thermal windows [29]. In laboratory rodents, thermal windows such as the ocular, auricular, dorsal or interscapular, and tail region have been used to assess stress [28,30] or pain [31]. For example, Lecorps et al. [32] found that eye temperature increased in mice undergoing an elevated plus maze test, while tail temperature diminished as a physiological response to stress (a result that was associated with anxiety-related behaviors). The ocular surface has great vascular sensitivity because the two main arteries responsible for its irrigation (the *arteria supraorbitalis* and *angularis occuli*), as well as the innervation through the facial nerve, rapidly respond to autonomous tone changes and endogenous catecholamines [29]. Likewise, Zevgolis et al. [33] reported that ocular IRT increased during the experimental handling of wild mice due to stress-induced hyperthermia (SIH). A similar response is observed in the auricular window, as shown by Wokke [34] in mice. In this study, restraining methods to administer IP drugs increased ear temperature and corticosterone levels. These temperature variations are mediated by sympathetic activity and vasodilation in the main blood vessels supplying irrigation (external jugular vein, external carotid artery, and its branches into marginal ear arteries) [29,35]. Hutu et al. [36] determined that IRT measured in the ear is positively correlated with rectal temperature in rabbits. Therefore, considering that SIH also causes changes in the amount of dissipated heat in thermal windows, ear temperature could be a way to assess acute stress.

For laboratory rodents, a thermal window that is closely related to sympathetic activation and norepinephrine (NE) release after the activation of the SAM axis is the interscapular region. In this zone, small mammals have large deposits of brown adipose tissue (BAT), a thermogenic structure whose activity depends on NE binding to β_3_-adrenoreceptors located in BAT [37]. The increased thermogenic activity of this tissue has been associated with corticosterone secretion and with the administration of β_3_-adrenoreceptor agonists and NE [38,39]. Furthermore, SIH is also related to BAT thermogenesis in rats and humans after excessive stress [40]. 

Lastly, the tail of rats is considered an important thermal window because it contributes to up to 25% of heat dissipation (by vasoconstriction) due to arteriovenous anastomosis (from the coccygeal artery) [29,41]. Vasoconstriction of peripheral regions such as the tail and paws is mediated by the sympathetic redistribution of blood flow to key organs (e.g., the heart and brain). In mice exposed to acute stressors, the superficial tail temperature decreased during different handling procedures, while the surface temperature of the body (assessed in the dorsal region of the mice) increased as a response to SIH [42]. Gjendal et al. [30] also reported a decrease in tail temperature (up to 3.5 °C) in mice exposed to three stressors (a maze test, IP injection, and isoflurane anesthesia) as a result of vasoconstriction of tail blood vessels. 

The current literature suggests IRT as a non-invasive complementary tool to assess well-being in animals, including stress-related responses [24,43]. Although there are some studies regarding IRT and the pre-slaughter or antemortem period in domestic species such as pigs [44,45], there is no study up to now where IRT has been used to evaluate the effect of euthanasia methods on laboratory species. Therefore, the present study aimed to assess the infrared thermal response of laboratory rats (*Rattus norvegicus*) during the application of six euthanasia methods to determine the method that prevents or diminishes the stress response and prolonged suffering. Injectable drugs (pentobarbital, ketamine + xylazine), inhalant agents (CO_2_, isoflurane), physical methods (decapitation), and the combination of inhaled and injectable anesthetics (CO_2_ + ketamine) were evaluated with IRT to identify changes in the surface temperature of four anatomical regions (ocular, auricular, interscapular, and caudal). Also, differences by sex according to the thermal window and the euthanasia method will be studied. The hypotheses of the study were as follows: (i) the use of IRT during different euthanasia methods will help to recognize changes in the surface temperature of laboratory rats (*R. norvegicus*) in response to stress perception related to the method and (ii) the combination of an injectable anesthetic overdose (ketamine) with CO_2_ exposure as the euthanasia method will reduce thermal alterations associated with stress.

## 2. Materials and Methods

### 2.1. Location and Ethical Statement 

The present study was conducted at the Animal Facility and Experimental Surgery Facility from the Biotechnological Research Sub-Department of the Instituto Nacional de Rehabilitación Luis Guillermo Ibarra Ibarra, Mexico City, Mexico. All procedures were approved by the Committee for the Care and Use of Laboratory Animals (INRLGII/CICUAL/014/2021) at the National Institute of Rehabilitation Luis Guillermo Ibarra-Ibarra.

The handling and care of the laboratory animals was in accordance with the Mexican norm for laboratory animals NOM-062-ZOO-1999, published by the Department of Agriculture, Rural Development, Fisheries and Alimentation [46]. All dead animals were disposed of by incineration following NOM-062-ZOO-1999. 

### 2.2. Animals and Housing Conditions

A total of 60 adult Wistar rats (*R. norvegicus*), 30 male and 30 female, were purchased from the Center for Research and Advanced Studies at the National Polytechnic Institute (CINVESTAV-IPN). The animals had an average weight of 311 ± 62 g at 8–10 weeks old (in puberty) and were obtained with an animal health certificate to ensure they were free of infectious pathogens (bacteria, viruses, and parasites). The sample size was calculated using G*Power 3.1.9.7 (Heinrich-Heine-Universität Düsseldorf, Düsseldorf, Germany). The total sample size was 48 animals, considering an α error probability of 0.05, confidence level of 95%, power (1-β error probability) of 0.90, and correction among repeated measures of 0.5 for six experimental groups with five measurements. 

According to the principles of the 3Rs [47], reduction was applied to the total number of animals used by reusing animals from finished protocols related to behavioral tests (e.g., balance beam or maze tests). Rats that were part of the control groups were selected to avoid the inclusion of animals undergoing invasive procedures or those with residual drug levels. Through a general physical examination, the animals were classified as healthy without signs of disease, stress, or pain-related behaviors. The physical exam considered body weight, posture, level of consciousness, secretions, the color of the mucosa, sneezing, and a species-specific behavioral repertoire. Rats showing signs of disease, injury, or pain were eliminated. Pregnant females were excluded.

Rats were housed in separate rooms according to sex. They were placed in groups of five animals per cage in standard polycarbonate cages for rats (47 × 36 × 21 cm) with wood shavings as bedding (Aspen, Nepco, Riverside, RI, USA) and without enrichment. The rats were maintained under a 12 h day–night cycle with lights on between 0500 h and 1700 h. The controlled temperature inside the housing room and the testing room was set at an average of 23.2 ± 0.5 °C and 22.9 ± 0.5 °C, respectively, with respective humidity levels of 48% and 52%. The rats had ad libitum access to food (LabDiet 5010, LabDiet, St. Louis, MI, USA) and purified water (in 500 mL drinking water bottles), and the cages were cleaned once a week. Visual health inspection was performed twice daily.

### 2.3. Experimental Design

**Figure 1:**
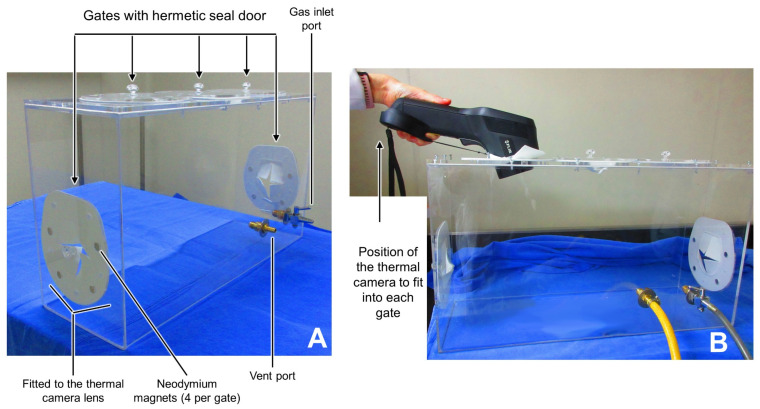
Customized acrylic euthanasia chamber for thermal imaging. (**A**) shows the components of the chamber, with the five hermetic seal doors and the respective gas inlet and vent port. (**B**) shows the position of the thermal camera that ensured the lens fit into each gate during the evaluation of inhalant euthanasia.

This was an experimental prospective–comparative study. All measurements were performed by a single trained and unblinded evaluator. Once the rats were selected for the study, they underwent habituation for 15 days to the customized euthanasia chamber, handling, and the evaluator’s presence. The animals were randomly divided into six groups by number generation (Microsoft Excel; Microsoft 365). A total of 10 rats were assigned in each group (5 males and 5 females) as follows: 

G_1_: Pentobarbital (Pentobarbital, Aranda^®^, Mexico City, Mexico) overdose at 400 mg/kg performed via IP injection with a 3 mL sterile syringe (Ambiderm^®^, Baja California, Mexico) following Lofgren et al. [48]’s procedure. The dose was calculated through a pilot study (no published data) using the minimal and maximal doses that appear in Reimer et al. [17]’s study. The selected dose resulted in rapid loss of consciousness, cardiorespiratory depression without excitation, loss of reflexes, and clinical death. G_2_: CO_2_ overdose administered inside a customized acrylic euthanasia chamber (Acrifactory, Mexico City, Mexico) (32.5 × 42 × 21 cm). The chamber had five gates with hermetically sealed doors that used neodymium magnets to avoid gas leaks. Each door was fitted to the size of the thermal camera lens to allow for thermal imaging (Figure 1). According to the AVMA [8], the flow rate was set at 30% of the chamber volume/min. G_3_: Decapitation using a rodent guillotine (51330, Senna, Mexico) [48]. G_4_: Inhalation of isoflurane (Fluriso, VET ONE^®^, Delhi, India) using the open-drop exposure method (two cotton swabs soaked with 2 mL of isoflurane each). The dose was calculated using the studies of Risling et al. [49] and de Brito [50] as a basis. The cotton swabs were placed where animals could not have direct contact with the inhalant anesthetic drug. G_5_: Ketamine (Ketamin-Pet, Aranda^®^, Mexico) + xylazine (Procin, Pisa Agropecuaria^®^, Nuevo México, Mexico) overdose administered at doses of 450 mg/kg IP and 45 mg/kg IP, respectively [8]. G_6_: Combination of ketamine (100 mg/kg IP) + CO_2_ (after 5–10 min of ketamine administration) [51]. 

Rats from all groups were assessed at five evaluation times. The basal time point represents assessment that was performed 24 h before the euthanasia method inside the housing room, and Ti_1_ represents three minutes before the application of the euthanasia in the test room. On the trial day, the rats were moved from the housing room to the test room, allowing for 30 min of rest and room acclimatization before starting the trial at Ti_2_, the time during the application of the method (e.g., while the animal received the IP dose of pentobarbital, ketamine + xylazine, or while it was inside the induction chamber or placed in the guillotine). Ti_3_ represents the time immediately after the application of the euthanasia method until loss of the righting reflex (LORR) as a sign of unconsciousness, and Ti_4_ represents the time until the cessation of breathing and heartbeat by visual assessment and assessment using a stethoscope (3M™ Littmann^®^ Classic III™, 3M, Saint Paul, MN, USA). The absence of palpebral, interdigital, and righting reflex was also used to confirm the euthanasia method. It is noteworthy to mention that all groups, except G_3_ (decapitation), had the same five evaluation times. In G_3_, the separation of the head from the body and unconsciousness was considered the same event; therefore, only four evaluation times were considered for this group (basal, Ti_1_, Ti_2_, and Ti_3_). 

### 2.4. Assessed Parameters

#### 2.4.1. Infrared Thermography (IRT) 

Thermal imaging was performed using an FLIR™ E60 camera (FLIR Systems, Orlando, FL, USA) positioned 1 m from the rats while maintaining a perpendicular angle to the subject (90°). Radiometric images were taken with an emissivity of 0.95, an IR resolution of 320 × 240 pixels, thermal sensitivity of <0.05 °C, and accuracy of ±2%. To prevent reflective heat affecting the acrylic cages placed in the housing room and the test room, the walls were covered with kraft paper. The handler used latex gloves when restraining the rats for IP injection and decapitation. Moreover, thermal imaging was performed at the same time of the day in all experimental groups (between 0800 h and 1500 h).

**Figure 2:**
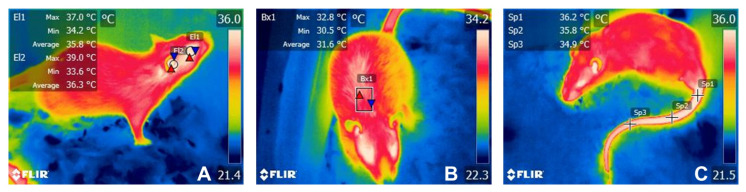
Representation of the four evaluated thermal windows. (**A**). T°_ocu_ was delimited by a circle (El1) covering the entire ocular region or ocular globe, without including the upper or lower eyelid. T°_ear_ was evaluated using a circle (El2) in the external ear canal to assess the irradiated temperature of the tympanic membrane and inner ear. (**B**). For T°_dor_, a rectangle (Bx1) was placed in the dorsal area over the interscapular space. (**C**) For T°_tai_, three spots (Sp1, Sp2, y Sp3) were placed at the proximal (T°prox), medial (T°medial), and distal (T°distal) segments of the tail.

During basal, Ti_1_, Ti_2_, Ti_3_, and Ti_4_, the four evaluated body regions (or thermal windows) were the ocular (T°_ocu_), auricular (T°_ear_), interscapular (T°_dor_), and tail (T°_tai_) regions. Thermal imaging for T°_ocu_ and T°_ear_ was taken from the right side of the animals. The delimitation of these regions of interest (ROIs) is shown in Figure 2. The thermal images were processed with FLIR Tools software (FLIR Systems, USA) to obtain the maximum, minimum, and average temperatures for T°_ocu_, T°_ear_, and T°_dor_. For T°_tai_, only the average temperature in the proximal, medial, and distal parts of the tail was recorded. This is due to the delimitation of the ROI with a spot, which only provides the average value. 

#### 2.4.2. Time to Death

To record the duration of each euthanasia method, after Ti_2_, the evaluator started a timer to register the time of death, the time to LORR, and the time to the cessation of breathing (visual assessment) and heartbeat (thoracic auscultation). The results were expressed as seconds, and the average value ± standard deviation (SD) of the 10 animals per group was recorded on an evaluation sheet.

### 2.5. Procedures

**Figure 3:**
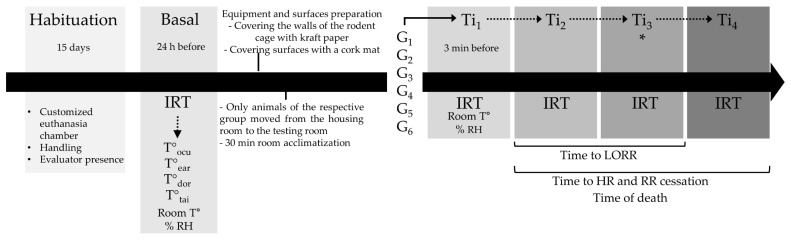
Experimental timeline for the euthanasia methods applied in rats. HR: heart rate; RR: respiratory frequency. * for G_3_, Ti_3_ includes LORR and HR/RR cessation.

After the 15-day habituation period and the random assignment of the rats into the six experimental groups, the procedures were performed as shown in Figure 3.

During the basal time point, IRT, as well as room temperature and relative humidity (% RH), was recorded inside the housing room of the selected experimental group with a wireless indoor and outdoor weather station with a hygrometer (Taylor^®^, Oak Brook, IL, USA). The equipment and surfaces were conditioned to IRT readings 24 h after by covering the walls of the polycarbonate cages with kraft paper and the use of wood shavings as bedding. The surfaces to place the guillotine, cages, and induction chamber were also covered by either sterile drapes or cork pads to avoid reflective heat. The rats from the corresponding group were moved from the housing room to the testing room so that euthanasia was not performed where the rest of the animals were housed. A period of 30 min was given to the rats to acclimatize them to the controlled temperature in the testing room and avoid stress related to transportation. Following this 30 min, the euthanasia method started. Room temperature, % RH, IRT, and time of death were recorded for each individual in all experimental groups. 

### 2.6. Statistical Analyses

All analyses were performed using the GraphPad Prism 10.0.0 (San Jose, Ca, USA) statistical package. The Shapiro–Wilk test was performed to establish data normality in the data set collected from T°_ocu_, T°_ear_, T°_dor_, and T°_tai_. Descriptive statistics were obtained and results were expressed as mean ± standard deviation (SD). A linear mixed model for repeated measures was used to evaluate the effect of the six euthanasia methods (treatments G_1_, G_2_, G_3_, G_4_, G_5_, and G_6_) at the five time points (basal, Ti_1_, Ti_2_, Ti_3_, and Ti_4_) for each of the four thermal windows. Multiple comparison of means was performed with the post-hoc Tukey test. In every case, the significance level was set at *p* < 0.05. The following statistical model was used:

Yijk = µ + τi + τj+ τiτj + βk + eij

where the symbols indicate the following:

Y = variable response (IRT);
τi = fixed effect (G_1_, G_2_, G_3_, G_4_, G_5_, G_6_);
τj = evaluation times (basal, Ti_1_, Ti_2_, Ti_3_, and Ti_4_);
β = aleatory effect (rat);
µ = general mean;
e = error.

To determine if there were differences between males and females from each experimental group, repeated measure ANOVA was performed with a Greenhouse–Geisser correction and a post-hoc Tukey test for multiple comparisons. Time of death, time to LORR, and time to the cessation of breathing (visual assessment) and heartbeat (thoracic auscultation) were expressed as mean ± SD. To establish the correlation between the thermal windows, Pearson correlation coefficients were calculated. All values with *p* < 0.05 were considered significant.

## 3. Results

Differences in the thermal response of the rats grouped in different experimental groups were obtained according to the thermal window, assessing their maximum (T°max), minimum (T°min), and mean temperature (T°mean). In general, G_2_ and G_4_ registered significant differences between evaluation times and between groups in three of the four thermal windows. Additionally, G_4_ individuals showed a progressive increase in temperature in all thermal windows, in contrast to the other experimental groups. 

### 3.1. Ocular Surface Temperature (T°_Ocu_)

Table 1 shows the mean and standard deviation (SD) values for the temperature of T°_ocu_. For T°max, T°min, and T°mean, differences among evaluation times were recorded in G_2_ (*p* = 0.0097) and G_4_ (*p* = 0.0001). For G_2_, a decrease in T°mean of up to 2.15 °C at Ti_4_ was observed when compared to basal values. Similarly, T°mean in G_4_ decreased by up to 5.82 °C at Ti_2_. Regarding differences between groups, a progressive temperature decline was registered in all euthanasia methods. However, differences between the groups were observed during Ti_2_ (*p* = 0.0007), Ti_3_ (*p* = 0.0002), and Ti_4_ (*p* = 0.0019), with the lowest T°mean values in G_2_ (33.79 ± 0.92 °C) and G_4_ (29.30 ± 1.23 °C) registered at Ti_2_.

### 3.2. Auricular Surface Temperature (T°_ear_)

Differences in T°mean between evaluation times were observed in G_4_ (*p* = 0.0011) (Table 2). When comparing Basal with Ti_2_, Ti_3_, and Ti_4_, a decrease in T°_ear_ by 5.82 °C, 5.04 °C and 4.77 °C, respectively, was reported. Between groups, G_2_ and G_4_ individuals had the lowest T°mean (30.80 ± 2.83 °C and 29.0 ± 1.76 °C, respectively) and differed from the other groups at Ti_2_ (0.0005), Ti_3_ (*p* = 0.001), and Ti_4_ (*p* = 0.04). 

### 3.3. Interscapular Surface Temperature T°_Dor_

Regarding T°mean of the interscapular region, differences between evaluation times were present in G_1_ (*p* = 0.007), G_2_ (*p* = 0.009), G_4_ (*p* = 0.001) and G_6_ (*p* = 0.004) (Table 3). G1 showed a difference of 1.4 °C when comparing Basal (32.46 ± 0.75 °C) vs. Ti_1_ (31.06 ± 0.82 °C), while a higher difference of 2.15 °C was obtained for G_6_ in Basal (32.21 ± 0.66 °C) vs. Ti_4_ (30.06 ± 0.67 °C). The inhalational agents recorded the lowest temperatures during Ti_2_ for G_2_ (29.08 ± 1.05 °C) and G_4_ (28.59 ± 1.28 °C), with temperature drops of 2.75 °C and 3.56 °C, respectively. Regarding differences by group, G_2_ and G_4_ significantly differed from the other four experimental groups at Ti_2_ (*p* = 0.0001), Ti_3_ (*p* = 0.001), and Ti_4_ (*p* = 0.0009). Particularly, G_4_ registered lower T°mean T°_dor_ than G_2_ in the mentioned evaluation times (28.59 ± 1.28, 28.78 ± 0.91 and 29.10 ± 0.72 °C, respectively). 

### 3.4. Tail Surface Temperature (T°_Tai_)

Table 4 shows the T°_tai_ at the proximal (T°prox), medial (T°medial) and distal segment (T°distal). In general, all groups showed a progressive decrease in the temperature, starting at Ti_2_. In the T°prox, G_1_ Ti_3_ and Ti_4_ significantly differed (*p* = 0.005) from Ti_1_ and Ti_2_, having a minimum temperature of 27.99 ± 1.23 °C at Ti_4_. Ti_2_ and Ti_3_ of G_3_ showed significant differences (*p* = 0.004) from Ti_1_, while G_4_ significantly differed (*p* = 0.002) at Ti_2_, Ti_3_ and Ti_4_. In the T°medial of G1, all events differed from Basal values (*p* = 0.001), while Ti_2_ and Ti_3_ of G_3_ were statistical different from Basal and Ti1 (*p* = 0.002). Similarly to the T°prox, T°medial (*p* = 0.02), and T°distal (*p* = 0.02) of G_4_ differed at Ti_2_, Ti_3_ and Ti_4_, recording the lowest values at Ti_2_ for both tail segments (25.70 ± 0.51 °C and 25.27 ± 0.50 °C, respectively). 

Between groups, significant differences were reported in Basal values of the three segments (*p* = 0.002, 0.001 and 0.005). Particularly, during Ti_2_ of the T°prox segment, differences were observed in G_2_ and G_4_ (*p* = 0.05), with the lowest T°_tai_ of 26.14 ± 2.02 °C and 26.42 ± 0.51 °C, respectively. 

Correlations between the four thermal windows and the experimental groups were obtained and are shown according to the experimental group in Supplementary Tables S1–S6. In all groups, significant (*p* < 0.001) and strong correlations (r > 0.96) were found between thermal windows. 

Table 5 summarizes descriptive analysis of the recorded times (in seconds) for each group. Considering the total time of death, the longest duration was observed in G_6_, followed by G_4_ (294.2 ± 74.3) and G_2_ (390.2 ± 171.4). Similarly, cessation of RR and HR was longer in G_6_ and G_4_. The groups that reached LORR faster were G_5_ and G_2_ (67 ± 10.3 and 78 ± 29.0 s, respectively). 

### 3.5. Effect of Sex on the Thermal Response of the Rats

**Figure 4:**
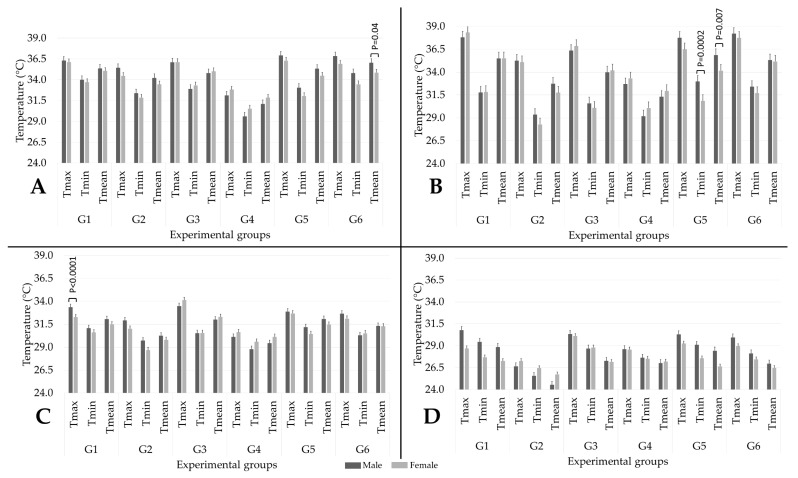
Effect of sex (5 males and 5 females in each group) on the maximum (max), minimum (min), and mean temperature of each thermal window in the six experimental groups: (**A**) T°_ocu_; (**B**) T°_ear_; (**C**) T°_dor_; (**D**) T°_tai_. G_1_: pentobarbital; G_2_: CO_2_; G_3_: decapitation; G_4_: isoflurane; G_5_: ketamine + xylazine; G_6_: ketamine + CO_2_.

Figure 4 illustrates the impact of sex on the temperatures of each thermal window according to the euthanasia methods. In general, no marked effect by sex was found in the present study. Only four statistically significant differences were registered for T°_ocu_, T°_ear_, and T°_dor_, while no effect was found on T°_tai_. For T°_ocu_ (Figure 4A). A significant difference (*p* = 0.04) was found in the Tmean of G_6_, where males had the highest temperatures (36.0 °C) in comparison to females (34.9 °C). However, a tendency to show a difference was observed in Tmax (*p* = 0.08) and Tmin (0.07). For T°_ear_ (Figure 4B), temperatures between males and females significantly differed in terms of Tmin and Tmean for G_5_ (*p* = 0.002 and *p* = 0.007, respectively), finding the highest temperatures in males rather than females (33.0 and 35.9 °C vs. 30.8 and 34.2 °C, respectively). Figure 4C shows a statistical significance between sexes in terms of Tmax for G_1_ (*p* < 0.0001) for T°_dor_. G_1_ males registered a Tmax T°_dor_ of 33.3 °C, while females recorded 32.2 °C. 

## 4. Discussion

Based on the findings of this study, in contrast to injectable, physical, and combined euthanasia methods, inhalant agents (CO_2_ and isoflurane) resulted in substantial alterations in the T°_ocu_, T°_ear_, T°_dor_, and T°_tai_ of Wistar rats. To date, there are no studies where IRT was used to evaluate different euthanasia methods or their effect on the thermal response of rats. However, studies addressing the mechanism of action of CO_2_ and isoflurane, as well as its overdose to induce euthanasia, have shown that both drugs trigger physiological stress and cardiovascular alterations in laboratory rodents [26]. Although both are considered safe, inexpensive, and effective methods to induce unconsciousness, the results obtained agree with previous findings suggesting that gases with anesthetic properties cause high levels of aversion and stress in mice and rats [15,52]. 

Before discussing the IRT results according to the thermal windows, the current literature regarding endocrine and behavioral changes after CO_2_ and isoflurane euthanasia in rats and mice suggests that the thermal response due to stress/distress could be associated with both methods. CO_2_ inhalation used in G_2_ rats induces hypercapnia, acidosis, and suppression of the synaptic potentials [52,53,54] and activates the stress-mediated sympathetic HPA and SAM axes [55]. This was corroborated by Borovsky et al. [56], who reported that exposure of rats to CO_2_ increased their blood pressure (50–60 mmHg) and NE concentration up to ten times in response to hypoxia. CO_2_ euthanasia at 10% is also related to distress due to a high exhibition of anxiety behavior in rats [11]. Moreover, aversion to high concentrations of CO_2_ (more than 40% of the induction chamber volume) is potentially associated with carbonic acid formation in the mucous membranes, causing irritation and discomfort behaviors such as spinning and pawing in rodents [57]. Acute exposure of mice to CO_2_, increased NE and adrenocorticotropic hormone (ACTH) levels [55], and studies in outbred mice and rats have shown that CO_2_ inhalation increases total serum protein levels, a biomarker associated with stress [58]. In contrast, Hackbarth et al. [59] found no behavioral signs of distress or endocrine alterations (ACTH and corticosterone) in rats undergoing CO_2_ euthanasia.

Studies comparing the effect of both CO2 and isoflurane euthanasia have also shown different results. Isoflurane administered in G_4_ rats is a volatile anesthetic that causes depression in the cardiorespiratory centers, leading to hypoxemia and death [26,60]. Although isoflurane has been considered as a method of CO_2_ euthanasia refinement [14,15], in comparison with CO_2_ and other volatile anesthetics, isoflurane has mild pungency [61], causing more aversion responses in laboratory rodents that is possibly due to airway irritation [52,62], air hunger, and dyspnea [52].

Powell et al. [57] found that the use of isoflurane during euthanasia increased anxiety-related behaviors, agitation scores, and corticosterone concentrations in mice compared to the low CO_2_ flow rate (30%), the same one used in the present research. Boivin et al. [63] compared isoflurane anesthesia followed by CO_2_, CO_2_, and barbiturates administration as euthanasia methods in mice. The authors found that, according to ACTH concentrations, barbiturates were less stressful than the other two methods. Nonetheless, cardiovascular alterations and pain/stress-related responses did not differ in the three methods, suggesting that isoflurane does not provide benefits above CO_2_ euthanasia. In this sense, Valentine et al. [64] reported that a combination of isoflurane and CO_2_ caused more signs of distress in mice and that CO_2_ alone has less evidence of stress in the animals.

In contrast to what was mentioned, Makowska and Weary’s [15] study reported that CO_2_ and inhalant agents are aversive to rodents, though the aversion is lower for isoflurane. Likewise, exposure to high concentrations of CO_2_ increased adrenaline and noradrenaline concentrations compared to isoflurane euthanasia. This could be indicative of a stress response, but since no stress-related behaviors (grooming, audible vocalizations) were reported in the CO_2_ group, CO_2_ could not be considered as more stressful than isoflurane [6]. In rats, Zardooz et al. [65] found that plasma corticosterone and insulin levels increased in rats exposed to CO_2_, while isoflurane caused a contrary reaction. In Hickman et al. [55]’s research, ACTH, corticosterone, and noradrenaline levels were detected in rats anesthetized with isoflurane; however, the increase was not as significant as with CO_2_.

The present study did not assess behavioral or endocrine parameters to associate the thermal response of rats to the different euthanasia methods. However, the literature shows that both methods trigger stress-related responses that have physiological consequences for the organism, which can be associated with temperature variations according to the thermal window. 

### 4.1. T°_ocu_

A significant increase in the T°mean of T°_ocu_ from Ti_2_ to Ti_3_ and from Ti_2_ to Ti_4_ in G_2_ and G_4_, respectively, was observed in the rats. The stress-mediated thermoregulatory impairment that CO_2_ and isoflurane cause on thermosensitive neurons due to the acidosis and hypoxic effect [66,67] could explain the increase in T°_ocu_. 

Several studies have shown that epinephrine, NE, ACTH, and corticosterone levels increase after CO_2_ and isoflurane exposure due to the potential stress that both drugs cause [6,68,69,70]. Although the present study did not consider these biomarkers for evaluation, their release modifies the vasomotor reaction of the microvasculature, inducing vasodilation in key organs (e.g., the eye) and an increased amount of dissipated heat, registered as higher IRT temperatures [29,71] like the ones observed in T°_ocu_ for G_2_ and G_4_ rats.

Ocular surface temperature in animals has been used as a thermal window to assess acute stress and pain, indicated by a recorded increase in both cases [72,73]. To the authors’ knowledge, there are only two studies combining IRT and the effect of isoflurane as an anesthetic [30,74], though these studies did not compare CO_2_ and isoflurane as a euthanasic. Gjendal et al. [30] determined that, from three different types of stimulus, isoflurane anesthesia in mice had a marked stress response due to the alterations in ocular temperature. Similarly, Vogel et al. [74] used isoflurane anesthesia and found that ocular temperature changed according to the isoflurane concentration and that this temperature also reflects rectal temperature in rodents. Nonetheless, no association was made with stress. 

Conversely, while there is no published evidence on euthanasia and ocular IRT, an increase in ocular temperature was reported in wild rodents (*Apodemus mystacinus*) as a reflection of SIH during the manipulation of individuals [33] during a fear-conditioned test in rats (increasing the eye temperature by up to 1.5 °C) [75], while in mice SIH and active behaviors were positively correlated [76]. Furthermore, in guinea pigs, the ocular temperature increased in relation to negative human interaction (petting) [77]. Similarly, Wongsaengchan et al. [78] used eye temperature to assess acute exposure to a stressor (small cage, handling, and restraint cone). The authors found significant increases in the left ocular temperature of females during restraint, together with corticosterone increases. 

The data suggest that the peripheral vasomotor changes might respond to the flight–fight response when exposed to a stressor. Increases in T°_ocu_ from Ti_2_ in all experimental groups suggest that rats perceived stress regardless of the euthanasia method. Nonetheless, knowing that CO_2_ and isoflurane inhalation might trigger stress-mediated pathways, this could explain the significant changes observed only in G_2_ and G_4_ from the application of the drug to LORR, probably due to an increased stress response. Finally, although both groups showed significant increases in T°_ocu_, G_2_ and G_4_ maintained overall lower temperatures than the rest of the groups, possibly due to heat loss facilitation due to the vasodilator properties of both drugs [53]. A similar result was obtained in Gjendal et al. [30]’s study, where isoflurane anesthesia in mice led to a reduction in T°max due to the hypothermia caused by general anesthetics.

### 4.2. T°_ear_

Comparable to T°_ocu_, significant increases in the T°mean of T°_ear_ from Ti_2_ were observed in both inhalant groups (G_2_ and G_4_). This pattern was expected because ear temperature assessed at the external ear canal is associated with the carotid artery and hypothalamic temperature, the main structure involved in central and peripheral thermoregulatory adaptations [79], particularly when exposed to stressors. Studies conclude that CO_2_ and isoflurane exert acute stress in rodents [52,55,58].

In animals, auricular temperature was associated with stress due to the administration of intraperitoneal drugs and restraining techniques in Wokke [34]’s study, as well as in rabbits during handling [80]. In rats, increases ranging between 0.8 and 1.5 °C were observed during conditioned fear reactions [75]. In the present study, in all experimental groups, an increase in the T°mean of T°_ear_ was observed. However, only CO_2_ and isoflurane caused significant increases. This response and its association with previously reported behavioral and endocrine responses with CO_2_ and isoflurane euthanasia/anesthesia might cause SIH. Since authors such as Hutu et al. [36] have concluded that superficial ear temperature is correlated to core temperature (around 37.1 ± 0.2 °C), the increase in T°_ear_ could be the reflection of SIH in G_2_ and G_4_. 

In contrast to the reported findings, some studies have not found significant changes or decreases in the ear temperature of mice and rats. This might be because of the lack of arteriovenous anastomosis present in other species, such as rabbits [81]. Additionally, conflicting results can be derived from the thermal window delimitation used by other authors (e.g., external ear canal or auricular pavilion). 

### 4.3. T°_dor_

An expected increase in T°mean values recorded for T°_dor_ after the administration of the euthanasia method was found in all experimental groups. Particularly, significant differences were reported in G_2_ and G_4_, maybe due to the induced acute stress that CO_2_ and halogenated anesthetics induce in rats. In the anatomical region where T°_dor_ was evaluated, large amounts of BAT can be found [37]. This thermogenic tissue responds to NE release. Borovsky et al. [56] and Hickman [55] reported NE increases after exposure of rats and mice to CO_2_ and isoflurane as potential stressors. 

Due to these elements the interscapular region was used in the present research to determine the effect of the different euthanasia methods, finding that G_2_ and G_4_ had significant increases in BAT activity. Similarly, a study by Blenkuš [42] reported the highest dorsal superficial temperatures in mice exposed to stressors (daily handling) and behavioral tests (voluntary interaction and elevated plus maze). Miyazono et al. [82] found that body surface temperature (assessed in the dorsal region of mice) increased after acute stress (e.g., reaction to a predator odor), while SIH is also related to BAT thermogenesis in rats and humans after excessive stress [40]. Pain perception in spinal lesion murine models has also shown increases in interscapular temperature, an effect that can be lessened with the administration of analgesic drugs [43]. 

Therefore, the data suggest that the significant local hyperthermia detected in T°_dor_ of G_2_ and G_4_ subjects could be perceived as a negative stimulus, particularly in both experimental groups.

### 4.4. T°tai 

A progressive reduction in T°_tai_ was observed for T°prox, T°medial, and T°distal, regardless of the experimental group. This is due to the vasoconstrictor effect of catecholamines on the microcirculation of peripheral regions such as the tail and paws, as well as the subsequent reduction in radiated heat detected by IRT [29,41].

In different studies, the superficial temperature of the tail has been used to assess stress and the emotional responses of laboratory rodents, where the effect is a reduction from basal values after the exposure of the stressor, as found in the present research. Exposure to stressors such as handling and restraint have been shown to reduce the tail temperature of rats [83]. Fear-conditioned rats registered a gradual decrease in tail surface temperature of up to 5.3 °C [75], while the temperature in the tail also decreased in mice during an elevated plus maze test as a result of stress and anxiety [32]. Likewise, Blenkuš et al. [42] reported decreases in tail temperature after 30–60 s of exposure to a stressor (daily handling, voluntary interaction test, and elevated plus maze test). Furthermore, Weitkamp [83] has mentioned that T°_tai_ can not only serve to identify acute stressors, but also provides insight into their intensity. This is relevant and consistent with the present results because, even though all euthanasia methods resulted in thermal changes associated with stress, only the inhalant agents caused significant effects in T°_tai_ and all thermal windows. 

Although a reduction in T°_tai_ was reported in all euthanasia methods (confirming that euthanasia elicits stress-related changes regardless of the method), significant decreases were observed in G_2_ and G_4_, and the lowest T°_tai_ values were recorded in both groups. This could be due to the potent vasodilation properties of CO_2_ and isoflurane [53,84], triggering a circulatory shift to restrict peripheral circulation (T°_tai_) and redirect the blood flow to central sites (T°_ocu_, T°_ear_, and T°_dor_). 

### 4.5. Effect of Sex on the Thermal Response of Animals during Euthanasia Methods

The results obtained when evaluating the effect that sex has on the thermal response showed, in general, no marked differences between males and females. In total, four statistical differences were found in G_6_ for T°_ocu_, G_5_ for T°_ear_, and G_1_ for T°_dor_, showing an inconsistency in the results. This agrees with what was reported by Zevgolis et al. [33] regarding the eye temperature of mice, where no differences by sex were found.

In contrast, Faraji and Metz [28] reported differences between male and female mice. Females exposed to rearing deprivation as a stressor exhibited increased superficial temperature in the head and the back and a decrease in tail temperature, while males did not have differences. Another study from the same authors concluded that rats also show temperature differences when evaluated through IRT and that the stress responses of males and females differ depending on the sex of the experimenters [85]. Likewise, apart from the differences reported between females and males, the temperature of a specific thermal window can also differ depending on the sex, as shown in a study where, according to IRT, females were prone to show an exacerbated stress response to restraint [78]. 

A possible explanation for the lack of significant differences between males and females in the present study could be due to the short period of evaluation used for each euthanasia method. Euthanasia times are (and must be) short so as to avoid high levels of stress. Although, as shown in Table 5, G_4_ (294.2 ± 74.3 s) and G_2_ (390.2 ± 171.4) were two of the three euthanasia methods with longer time of death, this time might not have permitted the finding of differences according to sex. Powell et al. [57] mention that rodents require at least two minutes of stressor exposure to increase corticosterone values in response to stress. Nonetheless, since IRT has not been previously evaluated during euthanasia methods considering both sexes, future research needs to consider these factors. 

### 4.6. Time of Death and Additional Findings

Regarding the time of death, time of LORR, and cessation of RR and HR, the times obtained in the six experimental groups are in accordance with previous studies evaluating time of death with pentobarbital [18], CO_2_ [86], decapitation [87], isoflurane [6], and ketamine + xylazine [8]. 

Lastly, a distinct pattern and pronounced difference between G_4_ and the rest of the experimental groups should be noted. T°_ocu_, T°_ear_, T°_dor_, and T°_tai_ for G_4_ rats showed a progressive increase in the surface temperature from Ti_2_ to the death of the animals, apart from recording the lowest temperatures from Ti_2_ to Ti_4_ when compared to the other five groups. In contrast, animals from the other groups, including G_2_, presented a temperature increase from Ti_2_ to Ti_3_ and a subsequent decrease in all thermal windows. This suggests that the anesthetic stress and physiological response triggered by isoflurane is more marked than that induced by other inhalant, injectable, and physical methods of euthanasia. The present results are in agreement with what other authors have stated regarding isoflurane as a refinement method for CO_2_ [52,62] and affirm that precautions should be taken when deciding to use isoflurane as a sole method for the humane killing of research animals. 

### 4.7. Limitations and Future Recommendations

The main limitation of the current study, and a field for complementary research using IRT aimed to evaluate euthanasia methods, is the lack of monitoring using physiological markers such as NE, ACTH, corticosterone, glucose, and other parameters (e.g., rectal temperature) that have been reported to increase their concentration during the application of different types of euthanasia [26,55]. Moreover, histological analyses could also help to identify the possible tissular changes associated with an inflammatory response to different drugs, providing additional information according to the euthanasia method. Additionally, analyzing the time of death, IRT response, and other biomarkers could help to understand the influence of the application speed and the thermal response of rodents. In the present study, the novel conception of an anesthesia induction chamber designed to allow for IRT readings during inhalant euthanasia is a valuable tool that might serve to further assess how euthanasia drugs, in combination with other physiological, endocrinal, and behavioral parameters, can contribute to the refinement of animal research. 

In this sense, an important finding of the present study that can be considered for future research as a refinement in euthanasia procedures in Wistar rats is the combination of injectable agents and CO_2_. As the results showed, contrary to the use of CO_2_ alone, the combination administered in G_6_ diminished the thermal alterations observed in G_2_. This could be due to the sedative properties of ketamine before CO_2_ exposure, antagonizing NMDA receptors, modulating neuronal activity, and reducing the discomfort sensation with CO_2_ [88,89]. This could prevent physiological responses due to induced hypoxia, acidosis, and stress-related changes [12].

Regarding the non-significant effect of sex in the thermal response of the subjects, the contrasting information between the present results and the published literature shows the complexity of using IRT as a tool to evaluate stress. For example, this variable and the other factors mentioned by Wongsaengchan et al. [78] (e.g., period of evaluation, sex, left/right side for the taking of thermal image) are important elements that need to be considered in further studies where IRT is intended to be used as a tool to improve the welfare of laboratory rodents. Similarly, the weight of rodents should also be considered when using IRT because the thermal response of animals might differ according to their energy reserves and metabolic activity (e.g., obesity in mammals is associated with increased depots of adipose tissue) [90]. Moreover, studies have shown that external traits such as coat color or type of fur can affect the amount of radiated heat [91]. In the present study we only used Wistar rats (white coat); however, when using IRT in other strains or species, these traits need to be addressed to objectively interpret thermal imaging.

Considering that IRT serves as a non-invasive method to assess the thermal response that can be associated with vasomotor changes due to sympathetic activation, IRT could be implemented as a complementary tool to evaluate stress under other conditions (e.g., heat stress). Likewise, pain assessment and even disease detection can be other fields where thermal imaging could be applied together with biomarkers and other technologies with the aim of improving laboratory animal welfare [24,29,30,31,73]. 

## 5. Conclusions

Based on the results obtained, it can be concluded that CO_2_ and isoflurane elicit stress-mediated thermal responses during rat euthanasia. In particular, isoflurane exposure might be a euthanasia method that causes potential distress, and this must be considered when deciding to use this drug as part of a euthanasic protocol. Refinement techniques such as the combination of ketamine + CO_2_ were shown to minimize the alterations observed with the sole use of CO_2_, but further research is required to perform a comprehensive evaluation of this alternative. Furthermore, the present study shows the usefulness of IRT as a non-invasive tool for the evaluation of euthanasia techniques and the thermal response of laboratory rodents. In this way, thermal imaging could be recommended together with other physiological, endocrinal, and behavioral parameters to assess and improve the welfare of research animals.

## Figures and Tables

**Table 1:** Effect of the six euthanasia methods, assessed at five evaluation times, on the maximum, minimum, and mean surface temperature (mean ± standard deviation, SD) of T°_ocu_ (°C) in Wistar rats.

	Groups	Basal	Ti_1_	Ti_2_	Ti_3_	Ti_4_	*p*-Value
T°max	G_1_ (*n* = 10)	36.62 ± 0.56 ^a,1^	35.85 ± 0.84 ^a,1^	35.88 ± 0.83 ^a,1^	36.44 ± 0.36 ^a,2,3^	36.25 ± 0.38 ^a,3^	*p* = 0.97
	G_2_ (*n* = 10)	36.03 ± 0.76 ^b,1^	35.93 ± 0.65 ^b,1^	34.10 ± 1.65 ^a,b,2^	34.90 ± 0.91 ^a,b,2^	33.78 ± 1.18 ^a,2^	***p* = 0.007**
	G_3_ (*n* = 10)	36.62 ± 0.39 ^a,1^	36.26 ± 0.56 ^a,1^	35.60 ± 1.25 ^a,1^	35.97 ± 0.52 ^a,2,3^	ND	*p* = 0.98
	G_4_ (*n* = 10)	36.46 ± 0.39 ^b,1^	35.82 ± 0.48 ^b,1^	29.76 ± 1.61 ^a,2^	29.9 ± 1.34 ^a,1^	30.41 ± 0.96 ^a,1^	***p* = 0.0001**
	G_5_ (*n* = 10)	36.52 ± 0.39 ^a,1^	36.66 ± 1.08 ^a,1^	36.50 ± 1.16 ^a,1^	36.83 ± 0.81 ^a,3^	36.54 ± 0.52 ^a,3^	*p* = 0.99
	G_6_ (*n* = 10)	36.88 ± 0.69 ^a,1^	36.18 ± 1.07 ^a,1^	36.19 ± 1.03 ^a,1^	36.80 ± 0.10 ^a,3^	35.85 ± 0.90 ^a,3^	*p* = 0.11
*p*-value		*p* = 0.89	*p* = 0.88	***p* = 0.0005**	***p* = 0.0006**	***p* = 0.0001**	
T°min	G_1_ (*n* = 10)	34.04 ± 0.90 ^a,2^	33.28 ± 0.88 ^a,1^	33.42 ± 0.56 ^a,3^	34.26 ± 0.59 ^a,3^	34.23 ± 0.47 ^a,3^	*p* = 0.49
	G_2_ (*n* = 10)	32.91 ± 0.77 ^a,1,2^	33.33 ± 0.47 ^c,1^	30.78 ± 1.22 ^a,1,2^	32.34 ± 0.97 ^b,c,2^	31.24 ± 1.24 ^a,b,2^	***p* = 0.0042**
	G_3_ (*n* = 10)	33.94 ± 0.55 ^a,1,2^	33.55 ± 0.68 ^a,1^	32.05 ± 1.06 ^a,2,3^	32.88 ± 1.01 ^a,2,3^	ND	*p* = 0.12
	G_4_ (*n* = 10)	32.73 ± 0.94 ^b,1,2^	31.93 ± 0.93 ^b,1^	28.35 ± 1.30 ^a,1^	28.50 ± 1.10 ^a,1^	28.87 ± 0.83 ^a,1^	***p* = 0.0001**
	G_5_ (*n* = 10)	32.49 ± 0.93 ^a,1^	32.45 ± 0.77 ^a,1^	32.66 ± 1.21 ^a,2,3^	32.70 ± 1.07 ^a,2,3^	32.47 ± 0.89 ^a,2^	*p* = 0.99
	G_6_ (*n* = 10)	34.48 ± 0.97 ^a,1,2^	33.64 ± 1.35 ^a,1^	34.09 ± 1.18 ^a,3^	34.69 ± 1.00 ^a,3^	33.78 ± 1.59 ^a,2^	*p* = 0.99
*p*-value		***p* = 0.0023**	*p* = 0.99	***p* = 0.0002**	***p* = 0.0007**	***p* = 0.0010**	
T°mean	G_1_ (*n* = 10)	35.58 ± 0.61 ^a,1^	34.75 ± 0.96 ^a,1^	34.88 ± 0.73 ^a,2,3^	35.48 ± 0.31 ^a,3,4^	35.36 ±0.38 ^a,3^	*p* = 0.99
	G_2_ (*n* = 10)	34.88 ± 0.71 ^a,b,1^	34.90 ± 0.47 ^b,1^	32.89 ± 1.44 ^a,b,1,2^	33.79 ± 0.92 ^a,b,2^	32.73 ±1.21 ^a,2^	***p* = 0.0097**
	G_3_ (*n* = 10)	35.51 ± 0.40 ^a,1^	35.10 ± 0.49 ^a,1^	34.15 ± 1.40 ^a,2,3^	34.88 ± 0.49 ^a,2,3^	ND	*p* = 0.98
	G_4_ (*n* = 10)	34.96 ± 0.62 ^a,1^	34.21 ± 0.54 ^a,1^	29.14 ± 1.46 ^b,1^	29.30 ± 1.23 ^b,1^	29.77 ± 0.88 ^b,1^	***p* = 0.0001**
	G_5_ (*n* = 10)	34.84 ± 0.46 ^a,1^	34.79 ± 0.94 ^a,1^	34.94 ± 1.20 ^a,2,3^	35.10 ± 0.86 ^a,3,4^	34.89 ± 0.66 ^a,2,3^	*p* = 0.99
	G_6_ (*n* = 10)	35.90 ± 0.77 ^a,1^	35.05 ± 1.25 ^a,1^	35.38 ± 1.18 ^a,3^	35.96 ± 0.47 ^a,4^	34.98 ± 1.23 ^a,3^	*p* = 0.67
*p*-value		*p* = 0.20	*p* = 0.24	***p* = 0.0007**	***p* = 0.0002**	***p* = 0.0019**	

^a,b,c^ different literals indicate significant differences (*p* < 0.05) between events (basal, Ti_1_, Ti_2_, Ti_3_, Ti_4_). ^1,2,3,4^ different numerals indicate significant differences (*p* < 0.05) between treatments (G_1_, G_2_, G_3_, G_4_, G_5_, G_6_). Bold *p*-values represent statistically significant differences between events and treatments. ND = not determined due to the experimental group. Treatments (G_1_: pentobarbital; G_2_: CO_2_; G_3_: decapitation; G_4_: isoflurane; G_5_: ketamine + xylazine; G_6_: ketamine + CO_2_). Evaluation times (basal: 24 h before the procedure; Ti_1_: three minutes before the procedure; Ti_2_: during the application of the euthanasia method; Ti_3_: immediately after the application until loss of righting reflex (LORR); Ti_4_: from LORR to cessation of heartbeat and breathing).

**Table 2:** Mean ± standard deviation (SD) of T°_ear_ (°C) values of the six euthanasia methods, assessed in five evaluation times, registering the maximum, minimum and mean surface temperatures.

	Groups	Basal	Ti_1_	Ti_2_	Ti_3_	Ti_4_	*p*-Value
T°max	G_1_ (*n* = 10)	38.42 ± 0.57 ^a,1^	37.70 ± 0.37 ^a,1^	37.41 ± 0.96 ^a,2^	38.47 ± 0.37 ^a,3^	38.35 ± 0.81 ^a,2^	*p* = 0.09
	G_2_ (*n* = 10)	36.06 ± 0.89 ^a,2,4^	36.63 ± 0.70 ^a,1^	33.80 ± 3.95 ^a,1,2^	34.72 ± 1.80 ^a,2^	32.64 ± 4.40 ^a,1,2^	*p* = 0.68
	G_3_ (*n* = 10)	36.68 ± 0.64 ^a,2,3^	37.26 ± 0.66 ^a,1^	36.03 ± 2.16 ^a,2^	36.44 ± 1.64 ^a,2,3^	ND	*p* = 0.95
	G_4_ (*n* = 10)	36.99 ± 1.16 ^b,1,3,4^	36.87 ± 0.97 ^b,1^	29.76 ± 2.10 ^a,1^	30.54 ± 2.08 ^a,1^	30.90 ± 1.21 ^a,1^	***p* = 0.0003**
	G_5_ (*n* = 10)	37.47 ± 0.70 ^a,1,3^	37.13 ± 1.37 ^a,1^	35.37 ± 2.37 ^a,1,2^	37.73 ± 1.41 ^a,2,3^	38.04 ± 0.89 ^a,2^	*p* = 0.20
	G_6_ (*n* = 10)	38.15 ± 0.71 ^a,1^	37.70 ± 0.58 ^a,1^	37.89 ± 0.87 ^a,2^	38.67 ± 0.48 ^a,3^	37.44 ± 1.38 ^a,2^	*p* = 0.13
*p*-value		***p* = 0.0119**	*p* = 0.1226	***p* = 0.0003**	***p* = 0.0001**	***p* = 0.0003**	
T°min	G_1_ (*n* = 10)	32.25 ± 1.27 ^a,1^	31.36 ± 0.91 ^a,1^	31.97 ± 1.57 ^a,2^	32.53 ± 0.88 ^a,3^	31.92 ± 1.04 ^a,3^	*p* = 0.92
	G_2_ (*n* = 10)	30.24 ± 0.94 ^b,2^	29.78 ± 1.15 ^a,b,1^	28.89 ± 3.58 ^a,b,1,2^	27.67 ± 1.43 ^a,b,1^	27.53 ± 1.30 ^a,1^	***p* = 0.03**
	G_3_ (*n* = 10)	30.19 ± 0.94 ^a,2^	30.22 ± 1.09 ^a,1^	29.77 ± 1.71 ^a,1,2^	31.17 ± 1.2 ^a,2,3^	ND	*p* = 0.87
	G_4_ (*n* = 10)	31.95 ± 0.99 ^b,1^	31.22 ± 0.75 ^b,1^	27.81 ± 1.15 ^a,1^	28.31 ± 1.50 ^a,1,2^	28.88 ± 1.06 ^a,1,2^	***p* = 0.0023**
	G_5_ (*n* = 10)	32.06 ± 0.83 ^a,1^	31.57 ± 1.73 ^a,1^	31.90 ± 1.97 ^a,1,2^	31.44 ± 1.81 ^a,2,3^	32.61 ± 1.57 ^a,3^	*p* = 0.82
	G_6_ (*n* = 10)	32.56 ± 1.22 ^a,1^	31.61 ± 0.97 ^a,1^	32.80 ± 1.34 ^a,2^	32.35 ± 0.74 ^a,3^	30.99 ± 1.98 ^a,2,3^	*p* = 0.59
*p*-value		***p* = 0.0004**	*p* = 0.4742	***p* = 0.0005**	***p* = 0.0010**	***p* = 0.0018**	
T°mean	G_1_ (*n* = 10)	35.85 ± 0.84 ^a,1^	35.0 ± 0.65 ^a,1,2^	34.70 ± 1.09 ^a,2^	36.24 ± 0.63 ^a,2^	35.77 ± 0.80 ^a,3^	*p* = 0.08
	G_2_ (*n* = 10)	33.60 ± 0.92 ^a,1^	33.78 ± 0.69 ^a,1^	30.80 ± 2.83 ^a,1,2^	31.60 ± 1.61 ^a,1^	31.49 ± 2.42 ^a,1,2^	*p* = 0.25
	G_3_ (*n* = 10)	34.08 ± 0.94 ^a,1^	34.32 ± 0.82 ^a,1,2^	33.55 ± 1.15 ^a,2^	34.40 ± 1.64 ^a,2^	ND	*p* = 0.91
	G_4_ (*n* = 10)	34.82 ± 0.54 ^a,1^	34.47 ± 0.92 ^a,1,2^	29.0 ± 1.76 ^b,1^	29.78 ± 1.90 ^b,1^	30.05 ± 1.20 ^b,1^	***p* = 0.0011**
	G_5_ (*n* = 10)	35.27 ± 0.75 ^a,1^	34.85 ± 1.45 ^a,1,2^	33.78 ± 2.23 ^a,2^	35.21 ± 1.67 ^a,2^	36.02 ± 1.22 ^a,1,2^	*p* = 0.43
	G_6_ (*n* = 10)	35.88 ± 0.99 ^a,1^	35.11 ± 0.62 ^a,2^	35.06 ± 2.26 ^a,2^	35.58 ± 2.04 ^a,2^	34.57 ± 1.85 ^a,2,3^	*p* = 0.98
*p*-value		*p* = 0.06	***p* = 0.02**	***p* = 0.0005**	***p* = 0.001**	***p* = 0.04**	

^a,b,c^ different literals indicate significant differences (*p* < 0.05) between events (Basal, Ti_1_, Ti_2_, Ti_3_, Ti_4_). ^1,2,3,4^ different numerals indicate significant differences (*p* < 0.05) between treatments (G_1_, G_2_, G_3_, G_4_, G_5_, G_6_). Bold *p*-values represent statistically significant differences between events and treatments. ND= not determined due to the experimental group. Treatments (G_1_: pentobarbital; G_2_: CO_2_; G_3_: decapitation; G_4_: isoflurane; G_5_: ketamine + xylazine; G_6_: ketamine + CO_2_). Evaluation times (basal: 24 h before the procedure; Ti_1_: three minutes before the procedure; Ti_2_: during the application of the euthanasia method; Ti_3_: immediately after the application until loss of righting reflex (LORR); Ti_4_: from LORR to cessation of heartbeat and breathing).

**Table 3:** Mean ± standard deviation (SD) of T°_dor_ (°C) maximum, minimum and mean values of the six euthanasia methods, assessed in five evaluation times.

	Groups	Basal	Ti_1_	Ti_2_	Ti_3_	Ti_4_	*p*-Value
T°max	G_1_ (*n* = 10)	33.28 ± 0.78 ^a,1^	32.04 ± 1.14 ^a,1^	33.07 ± 0.94 ^a,2^	32.57 ± 0.67 ^a,2^	33.07 ± 0.87 ^a,2^	*p* = 0.89
	G_2_ (*n* = 10)	32.88 ± 1.10 ^b,1^	31.47 ± 0.91 ^a,1^	30.41 ± 0.98 ^a,1^	31.37 ± 1.39 ^a,2^	31.20 ± 0.75 ^a,1^	***p* = 0.01**
	G_3_ (*n* = 10)	33.58 ± 0.84 ^a,b,1^	32.09 ± 1.57 ^a,1^	34.78 ± 0.82 ^b,2^	34.76 ± 1.14 ^b,3^	ND	***p* = 0.001**
	G_4_ (*n* = 10)	32.97 ± 0.94 ^b,1^	30.91 ± 0.78 ^a,1^	29.04 ± 1.21 ^a,1^	29.32 ± 1.01 ^a,1^	29.62 ± 0.66 ^a,1^	***p* = 0.0004**
	G_5_ (*n* = 10)	32.55 ± 0.62 ^a,1^	31.79 ± 1.22 ^a,1^	33.44 ± 0.76 ^a,2^	33.04 ± 1.05 ^a,2,3^	33.08 ± 0.51 ^a,2^	*p* = 0.09
	G_6_ (*n* = 10)	32.91 ± 0.72 ^b,1^	31.77 ± 0.55 ^a,1^	33.01 ± 1.30 ^a,2^	32.73 ± 1.02 ^a,b,2,3^	31.51 ± 1.06 ^a,1,2^	***p* = 0.06**
*p*-value		*p* = 0.16	*p* = 0.47	***p* = 0.005**	***p* = 0.002**	***p* = 0.008**	
T°min	G_1_ (*n* = 10)	31.51 ± 0.59 ^a,1^	30.32 ± 0.81 ^a,1^	30.35 ± 0.53 ^a,2^	30.75 ± 0.48 ^a,3^	31.23 ± 0.73 ^a,3^	*p* = 0.15
	G_2_ (*n* = 10)	30.98 ± 0.96 ^b,1^	29.54 ± 0.62 ^a,1^	28.89 ± 3.37 ^b,1^	28.13 ± 0.88 ^b,1^	28.39 ± 1.51 ^b,1^	***p* = 0.01**
	G_3_ (*n* = 10)	31.80 ± 0.56 ^a,1^	30.20 ± 0.65 ^a,1^	30.15 ± 1.97 ^a,2^	29.97 ± 2.43 ^a,2^	ND	*p* = 0.07
	G_4_ (*n* = 10)	31.27 ± 1.22 ^b,1^	29.55 ± 0.90 ^a,1^	28.17 ± 1.34 ^a,1^	28.30 ± 0.88 ^a,1,2^	28.64 ± 0.78 ^a,1^	***p* = 0.008**
	G_5_ (*n* = 10)	31.18 ± 0.49 ^a,1^	29.89 ± 1.51 ^a,1^	31.38 ± 1.36 ^a,3^	30.88 ± 1.16 ^a,3^	30.72 ± 0.50 ^a,2^	*p* = 0.50
	G_6_ (*n* = 10)	31.54 ± 0.61 ^c,1^	30.20 ± 0.74 ^b,1^	30.88 ± 0.70 ^b,2,3^	30.63 ± 1.37 ^b,2,3^	28.77 ± 0.73 ^a,1^	***p* = 0.007**
*p*-value		*p* = 0.73	*p* = 0.33	***p* = 0.01**	***p* = 0.02**	***p* = 0.003**	
T°mean	G_1_ (*n* = 10)	32.46 ± 0.75 ^c,1^	31.06 ± 0.82 ^a,1^	31.58 ± 0.73 ^a,b,2^	31.62 ± 0.41 ^a,b,c,3^	32.12 ± 0.75 ^b,c,2^	***p* = 0.007**
	G_2_ (*n* = 10)	31.83 ± 1.01 ^b,1^	30.58 ± 0.73 ^a,1^	29.08 ± 1.05 ^a,1^	29.35 ± 0.91 ^a,1,2^	29.24 ± 0.68 ^a,1^	***p* = 0.009**
	G_3_ (*n* = 10)	32.58 ± 0.62 ^a,1^	31.25 ± 1.03 ^a,1^	32.48 ± 0.93 ^a,2^	32.27 ± 1.05 ^a,3^	ND	*p* = 0.22
	G_4_ (*n* = 10)	32.15 ± 1.01 ^b,1^	30.21 ± 0.83 ^a,b,1^	28.59 ± 1.28 ^a,1^	28.78 ± 0.91 ^a,1^	29.10 ± 0.72 ^a,1^	***p* = 0.001**
	G_5_ (*n* = 10)	31.84 ± 0.53 ^a,1^	30.95 ± 1.27 ^a,1^	32.43 ± 1.05 ^a,2^	31.85 ± 0.97 ^a,3^	31.78 ± 0.41 ^a,2^	*p* = 0.07
	G_6_ (*n* = 10)	32.21 ± 0.66 ^c,1^	31.0 ± 0.61 ^a,b,1^	31.97 ± 0.64 ^b,c,2^	31.24 ± 1.06 ^a,b,c,2,3^	30.06 ± 0.67 ^a,1^	***p* = 0.004**
*p*-value		*p* = 0.57	*p* = 0.99	***p* = 0.0001**	***p* = 0.001**	***p* = 0.0009**	

^a,b,c^ different literals indicate significant differences (*p* < 0.05) between events (Basal, Ti_1_, Ti_2_, Ti_3_, Ti_4_). ^1,2,3,^ different numerals indicate significant differences (*p* < 0.05) between treatments (G_1_, G_2_, G_3_, G_4_, G_5_, G_6_). Bold *p*-values represent statistically significant differences between events and treatments. ND= not determined due to the experimental group. Treatments (G_1_: pentobarbital; G_2_: CO_2_; G_3_: decapitation; G_4_: isoflurane; G_5_: ketamine + xylazine; G_6_: ketamine + CO_2_). Evaluation times (basal: 24 h before the procedure; Ti_1_: three minutes before the procedure; Ti_2_: during the application of the euthanasia method; Ti_3_: immediately after the application until loss of righting reflex (LORR); Ti_4_: from LORR to cessation of heartbeat and breathing).

**Table 4:** Mean ± standard deviation of T°_tai_ (°C) values at the proximal (T°prox), medial (T°medial), and distal (T°distal) segments of the tail, assessed on the six euthanasia methods at five evaluation times.

	Groups	Basal	Ti_1_	Ti_2_	Ti_3_	Ti_4_	*p*-Value
T°prox	G_1_ (*n* = 10)	33.42 ± 1.76 ^b,1^	28.81 ± 3.2 ^a,1,2^	29.69 ± 2.52 ^a,1,2^	28.80 ± 1.97 ^b,1^	27.99 ± 1.23 ^b,1^	***p* = 0.005**
	G_2_ (*n* = 10)	28.09 ± 2.31 ^a,b,2^	28.59 ± 1.92 ^a,2^	26.14 ± 2.02 ^a,2^	26.35 ± 1.88 ^a,1^	25.71 ± 1.62 ^a,1^	*p* = 0.07
	G_3_ (*n* = 10)	31.30 ± 1.60 ^a,b,2^	31.61 ± 0.75 ^b,1^	29.18 ± 0.85 ^a,1,2^	28.82 ± 0.98 ^a,1^	ND	***p* = 0.004**
	G_4_ (*n* = 10)	31.70 ± 1.64 ^b,2^	30.74 ± 1.50 ^b,1,2^	26.42 ± 0.51 ^a,2^	26.75 ± 0.94 ^a,1^	27.36 ± 0.84 ^a,1^	***p* = 0.002**
	G_5_ (*n* = 10)	29.99 ± 0.65 ^a,2^	29.62 ± 1.28 ^a,1,2^	30.22 ± 2.01 ^a,1^	29.75 ± 1.62 ^a,1^	29.23 ± 1.50 ^a,1^	*p* = 0.53
	G_6_ (*n* = 10)	30.31 ± 1.28 ^a,2^	29.97 ± 2.11 ^a,1,2^	30.01 ± 2.25 ^a,1,2^	29.17 ± 2.31 ^a,1^	27.82 ± 3.28 ^a,1^	*p* = 0.79
*p*-value		***p* = 0.002**	***p* = 0.03**	***p* = 0.05**	*p* = 0.15	*p* = 0.06	
T°medial	G_1_ (*n* = 10)	33.49 ± 1.79 ^a,3^	27.09 ± 3.84 ^b,1^	28.82 ± 2.63 ^b1^	27.19 ± 2.04 ^b,1^	26.21 ± 1.48 ^b,1^	***p* = 0.001**
	G_2_ (*n* = 10)	26.75 ± 2.78 ^a,1^	27.88 ± 2.29 ^a,1^	25.80 ± 2.81 ^a,1^	25.11 ± 2.02 ^a,1^	24.56 ± 2.15 ^a,1^	*p* = 0.98
	G_3_ (*n* = 10)	30.48 ± 2.07 ^b,2^	30.65 ± 1.36 ^b,1^	27.03 ± 1.37 ^a,1^	26.83 ± 1.28 ^a,1^	ND	***p* = 0.002**
	G_4_ (*n* = 10)	30.65 ± 2.07 ^b,2^	29.54 ± 1.89 ^b,1^	25.70 ± 0.51 ^a,1^	25.92 ± 1.10 ^a,1^	26.06 ± 0.82 ^a,1^	***p* = 0.02**
	G_5_ (*n* = 10)	28.77 ± 0.91 ^a,2^	28.18 ± 1.75 ^a,1^	28.95 ± 3.15 ^a,1^	28.18 ± 2.56 ^a,1^	27.59 ± 2.30 ^a,1^	*p* = 0.78
	G_6_ (*n* = 10)	29.24 ± 1.95 ^a,2^	28.37 ± 2.38 ^a,1^	28.54 ± 2.47 ^a,1^	27.64 ± 1.48 ^a,1^	25.20 ± 2.05 ^a,1^	*p* = 0.09
*p*-value		***p* = 0.001**	*p* = 0.23	*p* = 0.37	*p* = 0.34	*p* = 0.41	
T°distal	G_1_ (*n* = 10)	33.34 ± 2.08 ^c,1^	26.50 ± 3.77 ^b,1^	28.13 ± 2.68 ^b,1^	26.54 ± 2.10 ^b,1^	25.83 ± 1.61 ^b,1^	***p* = 0.003**
	G_2_ (*n* = 10)	26.29 ± 2.55 ^a,b,3^	27.08 ± 2.77 ^a,1^	24.41 ± 2.34 ^a,1^	24.30 ± 2.23 ^a,1^	23.67 ± 1.90 ^a,1^	*p* = 0.20
	G_3_ (*n* = 10)	29.23 ± 2.63 ^b,1,2^	29.32 ± 1.73 ^b,1^	25.28 ± 1.56 ^a,1^	25.14 ± 1.48 ^a,1^	ND	***p* = 0.002**
	G_4_ (*n* = 10)	30.23 ± 2.14 ^c,1,2^	28.65 ± 1.90 ^b,1^	25.27 ± 0.50 ^a,1^	25.67 ± 1.26 ^a,1^	25.77 ± 0.78 ^a,1^	***p* = 0.02**
	G_5_ (*n* = 10)	28.26 ± 1.13 ^a,b,2^	27.35 ± 1.79 ^a,1^	28.04 ± 3.08 ^a,1^	27.22 ± 2.62 ^a,1^	26.86 ± 2.43 ^a,1^	*p* = 0.47
	G_6_ (*n* = 10)	28.32 ± 2.32 ^a,b,2^	27.48 ± 2.28 ^a,1^	27.61 ± 2.60 ^a,1^	26.37 ± 1.84 ^a,1^	23.89 ± 1.80 ^a,1^	*p* = 0.10
*p*-value		***p* = 0.005**	*p* = 0.74	*p* = 0.33	*p* = 0.65	*p* = 0.19	

^a,b,c^ different literals indicate significant differences (*p* < 0.05) between events (basal, Ti_1_, Ti_2_, Ti_3_, Ti_4_). ^1,2,3,^ different numerals indicate significant differences (*p* < 0.05) between treatments (G_1_, G_2_, G_3_, G_4_, G_5_, G_6_). Bold *p*-values represent statistically significant differences between events and treatments. ND = not determined due to the experimental group. Treatments—G_1_: pentobarbital; G_2_: CO_2_; G_3_: decapitation; G_4_: isoflurane; G_5_: ketamine + xylazine; G_6_: ketamine + CO_2_. Evaluation times—basal: 24 h before the procedure; Ti_1_: three minutes before the procedure; Ti_2_: during the application of the euthanasia method; Ti_3_: immediately after the application until loss of righting reflex (LORR); Ti_4_: from LORR to cessation of heartbeat and breathing.

**Table 5:** Comparison between recorded times (in seconds) according to the experimental group (mean ± SD).

Group	Time of Death (s)	Time of LORR (s)	Time of RR Cessation (s)	Time of HR Cessation (s)
G_1_	230.1 ± 42.4	94.2 ± 19.8	193.8 ± 28.8	230.1 ± 42.2
G_2_	294.2 ± 74.3	78 ± 29.0	211.8 ± 64.1	305.5 ± 76.3
G_3_	6.2 ± 4.0	ND	ND	ND
G_4_	390.2 ± 171.4	97.8 ± 49.5	288 ± 15.7	390.2 ± 17.1
G_5_	257.9 ± 30.1	67 ± 10.3	172.3 ± 25.6	257.9 ± 30.1
G_6_	420.3 ± 47.3	122.7 ± 21.8	291.8 ± 31.7	420.3 ± 47.3

G_1_: pentobarbital; G_2_: CO_2_; G_3_: decapitation; G_4_: isoflurane; G_5_: ketamine + xylazine; G_6_: ketamine + CO_2_; HR: heart rate; LORR: loss of righting reflex; ND: not determined; RR: respiratory rate; s: seconds; SD: standard deviation.

## Data Availability

The data presented in this study are contained within the article.

## Supplementary Materials

The following supporting information can be downloaded at: https://www.mdpi.com/article/10.3390/ani13182820/s1, Table S1: Correlations “Pentobarbital” (G_1_); Table S2: Correlations “CO_2_ overdose” (G_2_); Table S3: Correlation “Decapitation” (G_3_); Table S4: Correlations “Inhalation of isoflurane” (G_4_); Table S5: Correlation “Ketamine” (G_5_); Table S6: Correlations “Combination of ketamine + CO_2_” (G_6_).
