# Supplementary material for: Thermal Response of Laboratory Rats (Rattus norvegicus) during the Application of Six Methods of Euthanasia Assessed by Infrared Thermography
Source: Animals (Basel). 2023 Sep 5;13(18):2820. doi: 10.3390/ani13182820 (PMC10526081; doi:10.3390/ani13182820)
Supplement: Supplementary file 1 [file animals-13-02820-s001.zip › animals-2551597-supplementary.pdf]

Table S1. Correlations "Pentobarbital" (G_1)

	$T°_{ocu}$	$T°_{ear}$	$T°_{dor}$	$T°_{tai}$
$T°_{ocu}$	1.00<br>P<0.001	0.991<br>P<0.001	0.986<br>P<0.001	0.374<br>P<0.001
$T°_{ear}$	0.991<br>P<0.001	1.00<br>P<0.001	0.977<br>P<0.001	0.370<br>P<0.001
$T°_{dor}$	0.986<br>P<0.001	0.977<br>P<0.001	1.00<br>P<0.001	0.377<br>P<0.001
$T°_{tai}$	0.374<br>P<0.001	0.370<br>P<0.001	0.377<br>P<0.001	1.00<br>P<0.001

Table S2. Correlations "CO_2 overdose" (G_2)

	$T°_{ocu}$	$T°_{ear}$	$T°_{dor}$	$T°_{tai}$
$T°_{ocu}$	1.00<br>P<0.001	0.992<br>P<0.001	0.973<br>P<0.001	0.960<br>P<0.001
$T°_{ear}$	0.992<br>P<0.001	1.00<br>P<0.001	0.971<br>P<0.001	0.963<br>P<0.001
$T°_{dor}$	0.973<br>P<0.001	0.971<br>P<0.001	1.00<br>P<0.001	0.977<br>P<0.001
$T°_{tai}$	0.960<br>P<0.001	0.963<br>P<0.001	0.977<br>P<0.001	1.00<br>P<0.001

Table S3. Correlations "Decapitation" (G_3)

	$T°_{ocu}$	$T°_{ear}$	$T°_{dor}$	$T°_{tai}$
$T°_{ocu}$	1.00<br>P<0.001	0.989<br>P<0.001	0.965<br>P<0.001	0.988<br>P<0.001
$T°_{ear}$	0.989<br>P<0.001	1.00<br>P<0.001	0.955<br>P<0.001	0.989<br>P<0.001
$T°_{dor}$	0.965<br>P<0.001	0.955<br>P<0.001	1.00<br>P<0.001	0.972<br>P<0.001
$T°_{tai}$	0.988<br>P<0.001	0.989<br>P<0.001	0.972<br>P<0.001	1.00<br>P<0.001

Table S4. Correlations "Inhalation of isoflurane" (G_4)

	$T°_{ocu}$	$T°_{ear}$	$T°_{dor}$	$T°_{tai}$
$T°_{ocu}$	1.00<br>P<0.001	0.992<br>P<0.001	0.982<br>P<.0.001	0.991<br>P<.0.001
$T°_{ear}$	0.992<br>P<0.001	1.00<br>P<0.001	0.992<br>P<0.001	0.980<br>P<.0.001
$T°_{dor}$	0.982<br>P<0.001	0.992<br>P<0.001	1.00<br>P<0.001	0.970<br>P<0.001
$T°_{tai}$	0.991<br>P<0.001	0.980<br>P<0.001	0.970<br>P<.0.001	1.00<br>P<0.001

Table S5. Correlations "Ketamine" (G_5)

	**T°$_{ocu}$**	**T°$_{ear}$**	**T°$_{dor}$**	**T°$_{tai}$**
T°$_{ocu}$	1.00<br>P<0.001	0.994<br>P<0.001	0.976<br>P<0.001	0.986<br>P<0.001
T°$_{ear}$	0.994<br>P<0.001	1.00<br>P<0.001	0.986<br>P<0.001	0.988<br>P<0.001
T°$_{dor}$	0.976<br>P<0.001	0.986<br>P<0.001	1.00<br>P<0.001	0.987<br>P<0.001
T°$_{tai}$	0.986<br>P<0.001	0.988<br>P<0.001	0.987<br>P<0.001	1.00<br>P<0.001

Table S64. Correlations "Combination of ketamine + CO_2" (G_6)

	**T°$_{ocu}$**	**T°$_{ear}$**	**T°$_{dor}$**	**T°$_{tai}$**
T°$_{ocu}$	1.00<br>P<.001	0.983<br>P<0.001	0.986<br>P<0.001	0.978<br>P<0.001
T°$_{ear}$	0.983<br>P<0.001	1.00<br>P<0.001	0.983<br>P<0.001	0.990<br>P<0.001
T°$_{dor}$	0.986<br>P<0.001	0.983<br>P<0.001	1.00<br>P<0.001	0.988<br>P<0.001
T°$_{tai}$	0.978<br>P<0.001	0.990<br>P<0.001	0.988<br>P<0.001	1.00<br>P<0.001
